# Curcumin to Promote the Synthesis of Silver NPs and their Self-Assembly with a Thermoresponsive Polymer in Core-Shell Nanohybrids

**DOI:** 10.1038/s41598-019-54752-4

**Published:** 2019-12-03

**Authors:** Albanelly Soto-Quintero, Nekane Guarrotxena, Olga García, Isabel Quijada-Garrido

**Affiliations:** Instituto de Ciencia y Tecnología de Polímeros. Consejo Superior de Investigaciones Científicas (ICTP-CSIC) c/Juan de la Cierva, 3 E-28006 Madrid, Spain

**Keywords:** Nanoscale materials, Molecular self-assembly

## Abstract

This work presents a simple one-pot protocol to achieve core-doped shell nanohybrids comprising silver nanoparticles, curcumin and thermoresponsive polymeric shell taking advantage of the reducing properties of phenolic curcumin substance and its ability to decorate metallic surfaces. Silver nanoparticles were synthesized, via sodium citrate and silver nitrate addition into a boiling aqueous solution of curcumin, monomers and surfactant. Curcumin and sodium citrate promoted silver nucleation, acting as reducing and stabilizing agents. These curcumin-capped AgNPs enabled, after adding the radical polymerization initiator, the assembling of the growing polymer chains around the hydrophobic AgNP surface. The resultant core-doped shell nanohybrids exhibit plasmonic, luminescent and volume thermoresponsive properties, with improved possibilities to be used as successful therapeutic platforms. In fact, the possibility to nanoconfine the synergistic antioxidant, antiviral, antibacterial features of silver and curcumin in one bioavailable hybrid paves the way to promising applications in the biomedical field.

## Introduction

The exciting electronic, optical, catalytic and antimicrobial properties of metal NPs can be stymied by their tendency to aggregate. To overcome this issue and provide metallic nanoparticles with additional features, the hybridization with polymeric materials is a successful strategy. In this regard, polymer micro/nanogels exhibit high colloidal stability, great versatility in their chemical and topological composition, drug loading and functionalization capabilities^1^ that make them unique vehicles and reservoirs for metal nanoparticles^2,3^. Additionally, the permeability of the gel-system coating allows a controlled loading and release of bioactive molecules and drugs.

Furthermore, polymer microgels are frequently used as nanoreactors for the *in situ* synthesis of metallic NPs^4^, and small particles decorating the microgel are successfully formed^5^. However, the control over the metallic NP structure and distribution is rather difficult.

Core-shell hybrid nanogels usually face metal@polymer nanohybrid synthesis from a different point of view; and the most reported protocols are based on the polymer self-assembling around a preformed metal NP. The ligand exchange using polymers with functional groups able to attach the metal NP is a successful approach to attain thin polymer shells^6^. However to achieve polymer coating with tunable thickness, polymerization in heterogeneous media, as free radical precipitation polymerization (FRPP) is the most widely choice^7,8^. Since this technique relies on the precipitation of water insoluble growing polymer chain, a previous surface preparation of metal NP is required. Note that the organic polymer is not compatible with the anionic citrate ligand, commonly used to synthesize metal NPs, in aqueous medium^9,10^. Therefore a variety of strategies can be found in the literature, mainly for gold^7,8,11–15^, whereas works focused on achieving silver@polymer core-shell nanogels are scarce; despite the reliable properties of silver NPs in applications as ultrasensitive analysis of molecules through Surface Enhanced Raman Spectroscopy (SERS)^2,16^, catalysis^17^, cancer cell imaging^16,18^ and other applications related to their antimicrobial properties^19^.

Recently, we developed one-step protocol supporting core-shell hybrid nanogel synthesis, regardless of the monomer polarity. The strategy was based on the use of hydrophobic thiolated methacrylate monomer as compatible bridge between the as-synthesized Au@citrate surface and the growing polymer chains^14^. The modification of the metal surface with the hydrophobic compound led to effective polymer self-assembling around the inorganic core.

**Figure 1:**
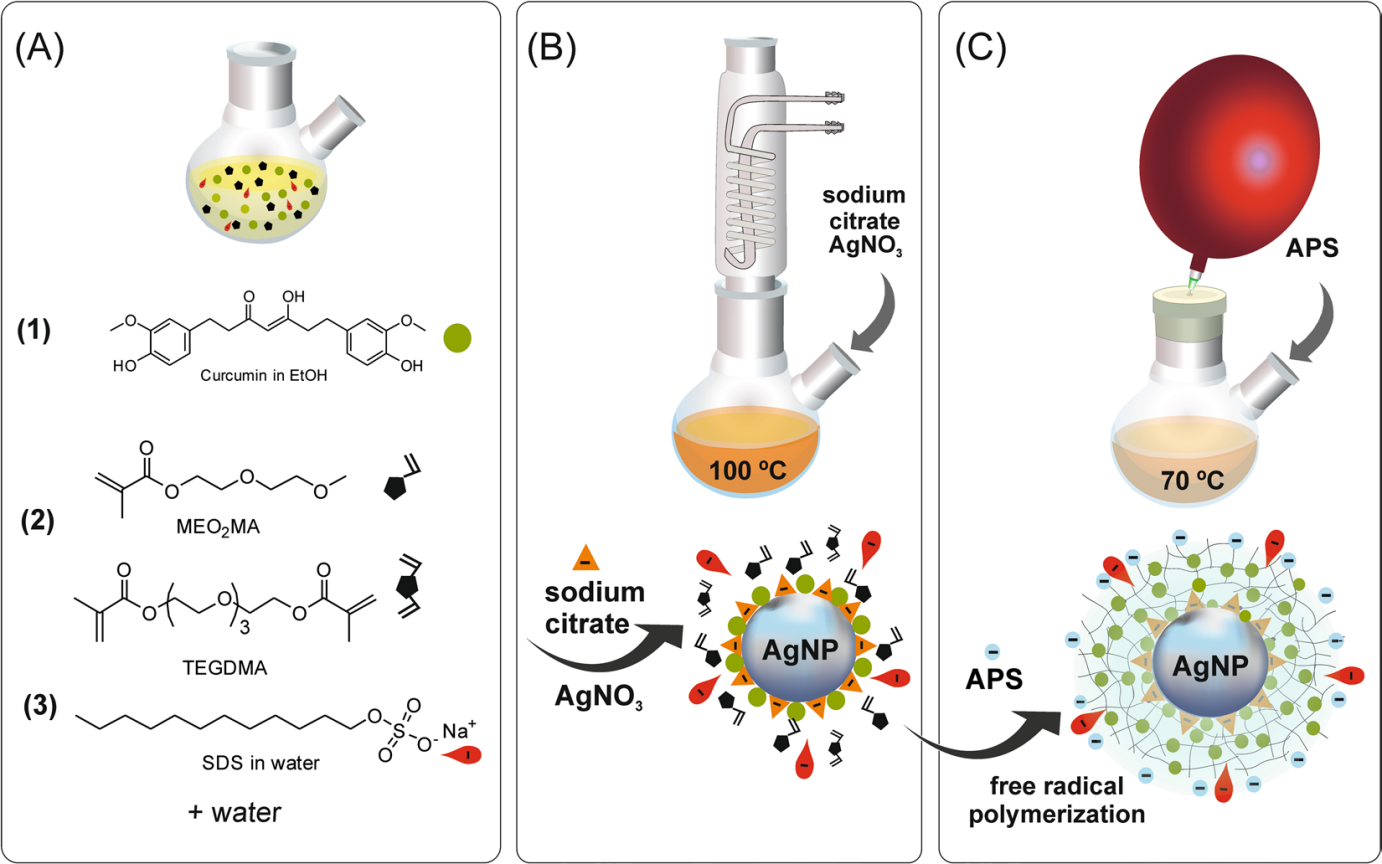
Schematic representation of the one-pot two-step chemical route to synthesize Ag@cur-P(MEO_2_MA) core-doped shell hybrid NPs. (**A**) Mixture at room temperature of curcumin, MEO_2_MA monomer, crosslinker and surfactant. (**B**) First step of AgNO_3_ reduction and Ag@cur NPs formation. (**C**) Free radical precipitation polymerization of MEO_2_MA and crosslinker encapsulating AgNPs forming Ag@cur-P(MEO_2_MA) NPs.

Thus, we envisioned, in this work, that hydrophobic molecules as curcumin (Fig. 1A), having a two-fold function, as reducing agent in silver NP synthesis as well as diminishing the interfacial energy between the growing polymer chain and the metallic surface, might trigger the core-shell hybrid NP assembly. Previous backgrounds evidenced the curcumin ability to reduce Ag^+^ and Au^3+^ ions to silver^20–22^ and gold^23^ nanoparticles, without any other additional reductant or in the presence of citrate and CTBA to modulate NP shape^24^. Thus, Kundu and Nithiyanantham^20^, in a pioneer work, were able to synthesize different shapes of AgNP (spheres, nanowires and anisotropic nanoflakes) by tuning the molar ratio of curcumin to AgNO_3_. Nevertheless, even though the reductant ability of curcumin has been already reported, it should be noted that, as far as we know, none has postulated that hydrophobic curcumin presence nearby metal surface may facilitate polymer self-assembling during post-crosslinking polymerization. In addition, by considering the antioxidant properties of this natural phenolic compound, our curcumin-promoted Ag@nanogel hybrid system will state a double benefit arising from the biomedical properties of curcumin itself, as antibacterial, self-healing^25,26^, antiviral^27^ and anti-inflammatory^28^; and from the antibacterial^19^ synergistic effect and remarkable antiviral^29^ activity of particles comprising silver nanoparticles and curcumin in one hybrid. Moreover, encapsulation of curcumin in the hydrophobic polymer shell will increase curcumin bioavailability protecting curcumin from hydrolytic degradation^30^, since this is the main limitation for its therapeutic applications.

## Experimental

### Materials

The monomer 2-(2-methoxyethoxy)ethyl methacrylate (MEO_2_MA) (Aldrich 95%) was purified by passing through a neutral alumina column. Tetraethylene glycol dimethacrylate (TEGDMA) (≥90%), trisodium citrate dihydrate (≥98%) and silver nitrate (AgNO_3_) (99.99%) were purchased from Aldrich. Sodium dodecyl sulfate (SDS) (≥97%) and ammonium persulfate (APS) (>98%), were purchased from Fluka. Curcumin (≥98%) was obtained from Acros Organics. All chemical reagents were employed as received. The solvents used, ethanol and water, were analytical and Milli-Q grade, respectively.

### Synthesis of Ag@cur-P(MEO_2_MA) core-doped shell hybrid NPs

Ag@cur-P(MEO_2_MA) hybrid nanoparticles, from now on also called as (Ag@cur-G), were synthesized through one-pot two-steps method (Fig. 1). Letter G denotes to thermoresponsive P(MEO_2_MA) polymer participation. A typical procedure for the synthesis is described below for sample Ag@cur-G4B (Table 1). In the first step (Fig. 1B), Ag@cur NPs (silver NPs decorated with curcumin (Table 1) were obtained by oxidation-reduction of sodium citrate, curcumin and AgNO_3_ in aqueous solution. The reaction was performed with 680 μL of curcumin solution (0.02 mmol in ethanol), MEO_2_MA monomer (1 mmol) and 272 μL of TEGDMA crosslinker solution (0.016 mmol in ethanol), 530 μL of SDS aqueous solution (9.15 10^–3^ mmol) and 20 mL of water at 100 °C in a 50 mL two-neck round bottom flask under stirring. The initial yellowish mixture (Fig. 1A) turned to more intense green-yellow color solution by the successive addition of 800 µL of sodium citrate (0.018 M, in aqueous solution) and 800 µL of AgNO_3_ (0.029 M, in aqueous solution) after 5 min of lapse-time in between, confirming AgNPs formation (Fig. 1B). The mixture was vigorously stirred for an additional 15 min at reflux and then allowed to cool down slowly to room temperature.

**Table 1:** Summary of curcumin concentration, *ζ*-potential, *Z* average diameter and polydispersity (PDI) by DLS, volume temperature induced phase transition (VPTT), swelling ratio (*Q*), size diameter by TEM and surface plasmon band wavelength (λ LSPR_max_) of Ag@cur-P(MEO_2_MA) core-doped shell hybrid NPs.

Entry	Curcumin^a^ wt%	*ζ* ^b^ (mV)	Z average (nm) DLS at 10 and 35 °C		PDI	VPTT (°C)	Swelling Ratio (*Q*)	Ag core Size (nm)	Surface plasmon wavelength (λLSPR_max,_ nm) at 10 and 35 °C	
Ag@cur^c^	3.15	−37.0	112.0	113.8	0.210	—	—	51	433	433
Ag@cur-G1A	1.05	−35.2	256.6	165.6	0.088	19.9	3.9	64	453	461
Ag@cur-G2A	2.10	−35.4	278.6	189.0	0.067	20.3	3.2	60	459	464
Ag@cur-G3A	3.15	−42.0	293.7	196.0	0.086	20.8	3.4	62	464	472
Ag@cur-G4A	3.80	−41.2	259.9	189.8	0.054	19.8	2.6	62	465	475
Ag@cur-G1B	1.05	−39.4	300.8	197.5	0.053	20.6	3.6	41	416	419
Ag@cur-G2B	2.10	−41.7	309.6	196.9	0.086	19.6	3.9	38	433	435
Ag@cur-G3B	3.15	−40.5	282.1	191.0	0.064	20.9	3.3	51	434	440
Ag@cur-G4B	3.80	−42.0	288.9	191.6	0.063	20.3	3.4	34	433	437

^(a)^Curcumin wt% is the feed curcumin/polymer ratio, ^(b)^ζ values were determined at 35 °C, ^(c)^Sample before (MEO_2_MA) polymerization (Fig. 1B).

In the second step (Fig. 1C), P(MEO_2_MA) shell formation around Ag@cur core was endowed by free radical precipitation polymerization (FRPP) of MEO_2_MA and TEGDMA crosslinker. The solution was degassed by N_2_ gas for 20 min at room temperature and polymerization was initiated by adding 500 μL of APS solution (0.045 M) after temperature was raised to 70 °C. The reaction was allowed to proceed for 2 h at 70 °C under stirring and N_2_ gas inlet (Fig. 1C). After that, the mixture was cooled down in an ice-cold water bath while it was exposed to the air.

The resultant core-doped shell NPs (Ag@cur-G4B) were purified by three centrifugation cycles by using successive decreasing centrifugal rates (8500, 6500 and 5000 rpm) at 18 °C for 10 min. Subsequent centrifugation rendered three supernatant fractions (f1, f2 and f3) and a fourth fraction (f4) corresponding to the final precipitate from the third cycle. This procedure ensured the complete removal of empty polymer particles and excess of reactants. Finally, NPs pellet was redispersed in Milli-Q water and kept at 4 °C in a refrigerator immediately prior to use.

During the synthesis, different reaction parameters such as reaction temperature (first step, Fig. 1B) and curcumin and sodium citrate concentration were considered in order to study their influence on size, shape and concentration of AgNPs. Ag@citrate NPs without curcumin, as control samples, were also synthetized.

### Synthesis of cur-P(MEO_2_MA) NPs or (cur-G)

The P(MEO_2_MA) crosslinked nanogels in the presence (cur-G) and absence (G0, control sample) of curcumin were synthesized through precipitation polymerization strategy in water. A typical procedure for the synthesis is described below for sample cur-G4 (Table 2). In a Pyrex tube equipped with a magnetic stirrer, 170 μL of a solution of curcumin in ethanol (0.005 mmol), MEO_2_MA monomer (0.25 mmol), 68 μL of TEGDMA crosslinker solution in ethanol (0.004 mmol), 132 μL of SDS aqueous solution (2.3 10^–3^ mmol) and 4 mL of Milli-Q water were added. After 20 min N_2_ gas purge, polymerization was initiated by heated up to 70 °C, followed by addition of 100 μL of APS solution (0.045 M). After around 10 min, the solution turned cloudy, indicating that polymerization started, and the solution was left to react for 2 h. To stop the reaction, the solution was cooling down in an ice-cold water bath while the tube was opened to air. Afterward, the sample was purified by centrifugation (10000 rpm, 45 min, 22 °C) and then the pellet redispersed in water. The final product was stored at 4 °C until further use.

**Table 2:** Summary of curcumin concentration, *ζ*-potential, Z average diameter and polydispersity (PDI) by DLS, volume temperature induced phase transition (VPTT), swelling ratio (*Q*), and maximum of curcumin absorbance (λ_abs_) of cur-P(MEO_2_MA) nanogels.

Entry	Curcumin^a^ wt%	*ζ*^b^ (mV)	Z average (nm) DLS at 10 and 35 °C		PDI	VPTT (°C)	Swelling Ratio (*Q*)	λ_max_ (nm) at 10 and 35 °C	
G0	0	−41.1	143.8	68.3	0.074	20.7	9.5	—	—
cur-G1	1.05	−43.6	122.0	73.4	0.093	20.0	4.67	425	422
cur-G2	2.10	−40.7	117.7	76.6	0.095	17.9	3.60	426	429
cur-G3	3.15	−43.1	114.4	79.5	0.124	17.0	3.00	424	425
cur-G4	3.80	−54.0	97.42	69.0	0.116	18.7	2.78	424	426

^(a)^Curcumin wt% is the feed curcumin/polymer ratio, ^(b)^ζ values were determined at 35 °C

### Methods for Characterization

FTIR spectra were recorded by Perkin Elmer Spectrum Two spectrometer using Attenuated Total Reflectance (ATR) accessory. UV-Vis absorption spectra, at controlled temperature, were carried out in a Cary 3 BIO-Varian UV-Vis spectrophotometer equipped with a Peltier temperature control device. The particle size and Zeta potential (ζ) of core-shell NPs and AgNPs were determined by a Zetasizer Nano ZS instrument (Malvern Instruments Ltd, UK) equipped with a 4 mW He–Ne laser operating at a light source wavelength of 633 nm and a fixed scattering angle of 173° for detection. Malvern Dispersion Software was used for data acquisition and analysis, applying the general purpose algorithm for calculating the size distribution. Transmission electron microscopy (TEM) images were recorded with a field emission scanning electron microscope (FESEM) Hitachi SU-8000 operated at 30 kV in transmitted electron imaging mode (S-TEM). Energy-dispersive X-ray spectroscopy (EDX) analysis to determine the elemental composition of the hybrid samples was performed using a Bruker Nano with X-Flash Detector 5030 coupled to the SEM. Data were recorded at an accelerating voltage of 15 kV.

The progress of sample purification by centrifugation was verified by UV-Vis spectroscopy (NanoDrop One Thermo-Scientific spectrometer). The centrifugation was performed by refrigerating micro centrifuge 5430 R (Eppendorf™) to obtain Ag@cur-G and cur-G nanoparticle pellets.

To determine the luminescence features, UV–Vis absorption and fluorescence spectra were recorded on a Perkin Elmer Lambda-35 and Perkin Elmer LS5OB spectrophotometer, respectively. Emission spectra of dilute solutions of Ag@cur-G core-doped shell nanohybrids and cur-G nanogels in water and dilute solutions of fluorescein in NaOH 0.1 M were recorded keeping absorbance at the excitation wavelength (λ_exc = _430 nm) less than 0.10. Quantum yields were obtained by comparing the studied samples with fluorescein standard by the following equation applicable for dilute solutions.

1 $${{\Phi}}_{F}=\frac{{I}_{A(s)}{I}_{E}{\eta }^{2}}{{I}_{A}{I}_{E(s)}{{\eta }_{(s)}}^{2}}{{\Phi}}_{s}$$

where *Φ*_F_ and *Φ*_s_ are the photoluminescence QY of the sample and that of the standard fluorescein (Φ_s_ = 0.925)^31^, respectively; *I*_E_ and *I*_E(s)_ are the integrated intensity of the emission curves corresponding to the sample and standard; *η* and *η*_(s)_ are the refractive indices of the sample and reference solution; and *I*_A_ and *I*_A(s)_ are the fraction of light absorbed by the sample and standard respectively estimated as:

2 $${I}_{A}=1-{10}^{-A}$$

where *A* is the absorbance at *λ*_exc_.

## Results and Discussion

### Synthesis and characterization of Ag@cur-P(MEO2MA) core-doped shell nanohybrids

The Ag@cur-P(MEO_2_MA) core-doped shell hybrid NPs were prepared by free radical precipitation polymerization (FRPP) of the stimuli-responsive MEO_2_MA monomer in the presence of TEGDMA (crosslinking agent), using curcumin-decorated Ag@cur NPs as seeds (Fig. 1). The presence of SDS guaranteed the control of NP size.

Among all the reagents involved in the process, silver nitrate and sodium citrate followed standard concentration procedures^32,33^. However, the dual key-role of curcumin, as reducing agent and growth-polymerization promoter in this specific synthesis, required additional investigation to understand and optimize the chemical variables (solubility, concentration and reaction temperature) in order to achieve homogeneous, monodisperse and mononuclear Ag@cur-P(MEO_2_MA) core-shell nanohybrids.

Indeed, FRPP is a useful and common strategy to synthesize particles of thermoresponsive polymers, as long as monomers are soluble at the reaction temperature and polymer precipitates when their chains reach a critical size. Then, since water solubility of curcumin is rather poor; pure curcumin compounds needed to be first dissolved into an organic phase of ethanol, MEO_2_MA monomer and crosslinker (Fig. 1A). A subsequent injection of aqueous solution of SDS surfactant followed by dropwise addition of water resulted in a good dispersion of monomers and curcumin. A color change from cloudy (at room temperature) to transparent yellowish dispersion (at the reaction temperature), indicated that both curcumin and monomers had been dissolved. Note that curcumin solubility increases with temperature^34^.

When the desired temperature was reached, sodium citrate solution was added to the reaction mixture already containing curcumin; and finally, in the presence of the two reducing agents, silver nitrate was added to the reaction (Fig. 1B).

AgNPs synthesis (outlined in Fig. 1B) was completed at two different temperatures, 90 °C and 100 °C (series -A and -B in Table 1, respectively). After 15 min of reaction, the flask was removed from the silicon bath and allowed to cool to room temperature. The as-synthesized silver nanocores had an average size around 60 nm (90 °C, series -A) and 40 nm (100 °C, series B) in diameter, respectively. To elucidate the role of curcumin as stabilizing agent, the sample obtained in this first step was purified by centrifugation to eliminate the excess of reagents that are not protecting the silver core. The presence of curcumin close to the AgNP surface, attained in this first step, was confirmed by ATR-FTIR spectroscopy (Fig. S1). As it can be observed in this Figure, the spectrum corresponding to Ag@cur (green line) is very similar to that of curcumin (blue line). The most prominent vibration band at ∼1510 cm^−1^ assigned to highly mixed vibration including C=O stretching^35^ appears in both spectra as well as other characteristic vibration bands of curcumin at 1626 cm^−1^(mixed C=C and C=O stretching), 1429 cm^−1^ (deformation of CH_3_), 1271 cm^−1^ (deformation of phenyl rings), 817 cm^−1^ (C-H out‐of‐plane aromatic motions). In this Fig. S1, spectra corresponding to sodium citrate and Ag@citrate NPs are also displayed. As it can be seen, two strong bands corresponding to the asymmetric and symmetric stretching of CO_2_^−^ groups at 1562 cm^−1^ and at 1387 cm^−1^, respectively appear with nearly similar intensity for the free sodium citrate spectrum (violet line); whereas for Ag@citrate spectrum (orange line), the most prominent band corresponds to the symmetric stretch at 1362 cm^−1^. In the spectrum corresponding to Ag@cur NPs (green line), this vibration band at 1362 cm^−1^ is absent and only the asymmetric stretching (∼ 1590 cm^−1^) can be hardly intuited overlapped with vibration bands from curcumin (blue line); these facts indicate that the affinity of sodium citrate for the silver surface is lower than that of curcumin. In addition, the bands attributed to C-H (2937–2948 cm^−1^) and C=O stretching vibrations of MEO_2_MA appear very weak, also suggesting a low interaction of the monomer with the silver surface.

Figure 1C outlines the polymer shell preparation. This second step was done without previous Ag@cur sample purification. For the purpose of thermoresponsive-shell polymerization, Ag@cur was first flushed with N_2_ for 20 min at the lowest possible temperature (room temperature), with the aim of reducing water evaporation during nitrogen flow. Subsequently, APS initiator was added and incubated for 2 h in a closed flask under stirring at 70 °C. The presence of hydrophobic curcumin nearby the metallic surface led to precipitation-polymerization of P(MEO_2_MA) around the AgNPs previously formed (Fig. 1B,C); and the resulting Ag@cur-P(MEO_2_MA) nanoparticles were born negatively charged due to the persulfate groups from the APS initiator, which promotes their colloidal stability. The successful encapsulation of Ag@cur NPs within P(MEO_2_MA) nanogels can be anticipated by the ATR-FTIR spectrum shown in Fig. S1 corresponding to f4 of Ag@cur-G3B sample (Table 1), which displays typical vibration bands attributed to both curcumin and P(MEO_2_MA). It should be noted that an attempt to encapsulate AgNPs synthesized solely using sodium citrate caused irreversible aggregation in the second polymerization step (Fig. S2). This fact evidences the role of hydrophobic curcumin to provide both a protection to the AgNPs and a good interface between the polymer and the metal surface.

Well-defined Ag@cur-P(MEO_2_MA) nanohybrids (Table 1) were obtained by selective removal of the silver-free nanogels by centrifugation (see Experimental Section). It should be taken into account that the number of empty polymer particles created during the polymerization process exceeded the number of AgNPs in the solution by about two orders of magnitude; and they should be removed from.

**Figure 2:**
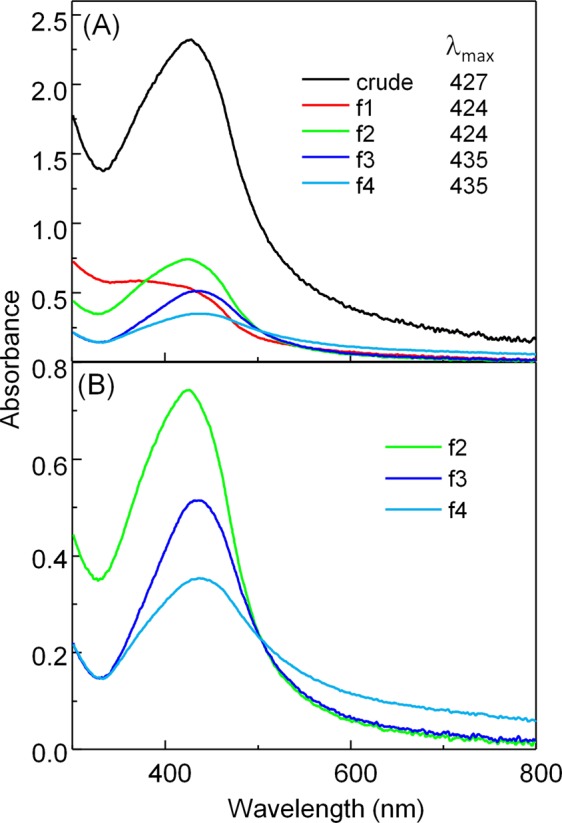
UV-Vis absorption spectra in (**A**) correspond to the four different fractions obtained from the centrifugation of Ag@cur-G4B crude (black line), f1 is the supernatant of the 1^st^ centrifugation (red line), f2 is the supernatant of the 2^nd^ centrifugation (green line), f3 is the supernatant of the 3^rd^ centrifugation (navy blue) and f4 is the final precipitate. In part (**B**) magnification of f2, f3 and f4 absorption spectra are presented.

As illustration, UV-Vis spectra at different purification steps by centrifugation of the Ag@cur-G4B crude sample are displayed in Fig. 2. Initially, the spectrum of the crude shows an intense band at 427 nm resulting from a combined contribution of curcumin absorbance and surface plasmon resonance (SPR) due to the collective oscillations of conductions electrons of silver NPs. Note that SPR maximum for spherical AgNPs can be typically tuned from 400 to 500 nm depending on their NP size and shape as well as their surrounding dielectric medium. Then, after the first and second centrifugation cycles, the supernatant fractions (f1 and f2 respectively) revealed absorption maxima around 424 nm, indicating that the most predominant absorbance is due to curcumin embedded in P(MEO_2_MA) NPs.

The third centrifugation cycle, however, on the basis of the supernatant fraction (f3) and its corresponding precipitate (f4) evidenced only core-doped shell Ag@cur-P(MEO_2_MA) nanoparticles content (absorption maxima at 435 nm). Therefore, the core-doped shell nanohybrids used for further characterization and analysis correspond to f4 fraction from each crude sample (Table 1).

Moreover, curcumin trapped-polymeric nanogels (cur-P(MEO_2_MA)) were synthesized through a similar precipitation-polymerization strategy reported for polymer encapsulating of Ag@cur (Fig. 1C, Ag@cur-P(MEO_2_MA)) and using similar curcumin/polymer ratios (see Experimental section and Table 2).

**Figure 3:**
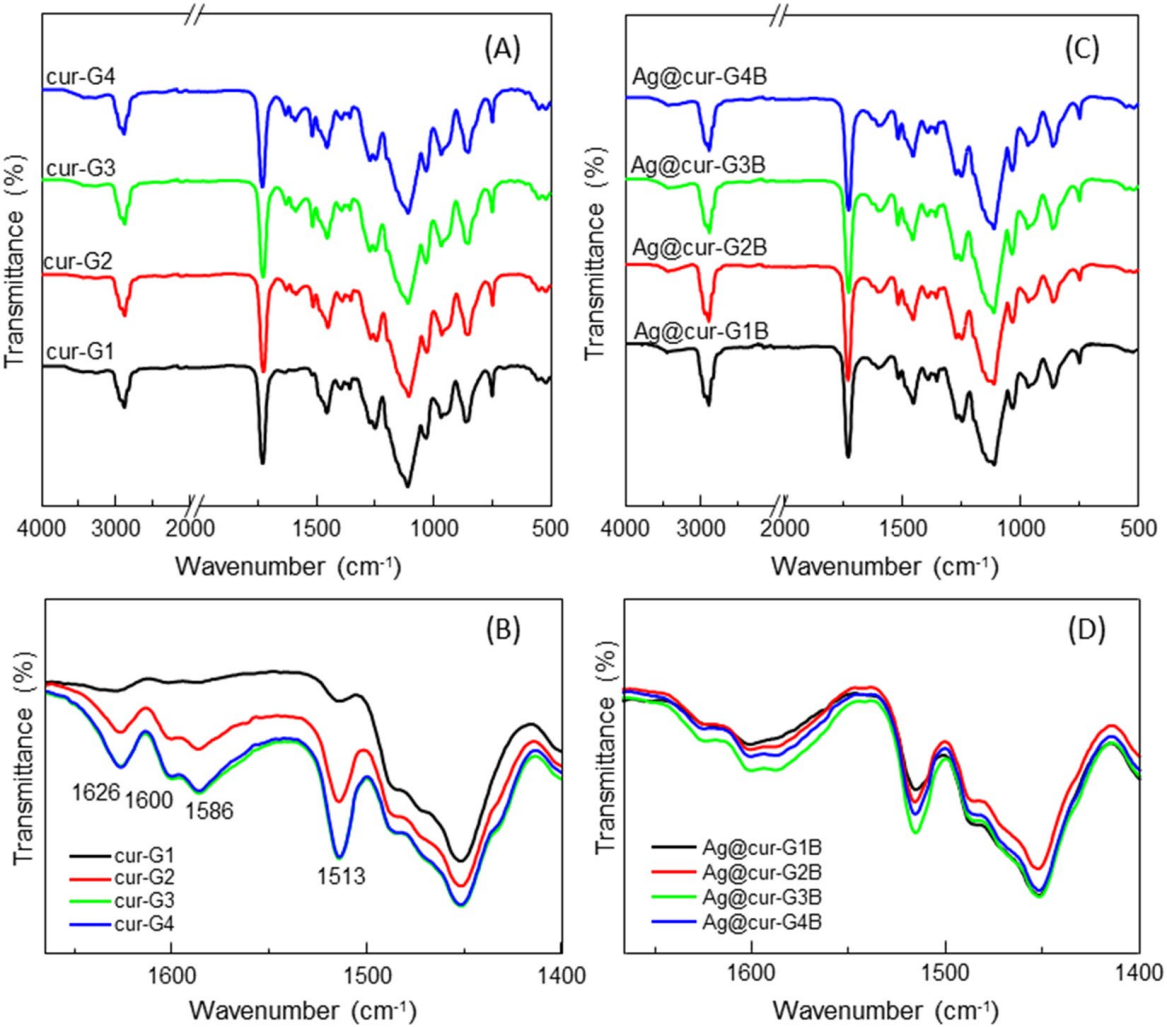
ATR-FTIR spectra, at different magnifications, corresponding to (**A,B**) cur-P(MEO_2_MA) nanogels (samples cur-G1 to -G4) and (**C,D**) Ag@cur-P(MEO_2_MA) core-doped shell hybrid NPs (samples Ag@cur-G1B to -G4B) at different curcumin concentrations indicated in Tables 1 and 2.

The chemical structure of both, polymeric NPs (cur-G) and hybrid NPs (Ag@cur-G) can be elucidated from the ATR-FTIR spectra shown in Fig. 3. Typical vibration bands (C=O stretching at 1725 cm^−1^ and C-O stretching at 1108 cm^−1^) corresponding to P(MEO_2_MA) are present in both types of NPs, in addition to vibration bands at 1626, 1600, 1586 and 1513 cm^−1^ attributed to curcumin molecule. Surprisingly, when comparing both cur-G and Ag@cur-G NPs, for similar curcumin/polymer ratios (Tables 1 and 2), qualitative differences were observed in their respective spectra evolution (Fig. 3). Consistently with curcumin data (Tables 1 and 2), curcumin related vibration bands seemed to be differently affected by its content. Cur-G series exhibited an increase tendency of intensity (Fig. 3A,B); whereas Ag@cur-G series (Fig. 3C,D) barely varied. This observation could be explained through the key-implication of curcumin in our nuclei-growth approach (Fig. 1) which determines, at the end, the morphology of Ag@cur-G nanohybrids. In fact, the specific silver-nucleation strategy (Fig. 1B) makes feasible that certain amount of curcumin be preferably confined at the metal surface, being subsequently enclosed at higher concentration during P(MEO_2_MA) polymer growing shell onto silver core NPs (Ag@cur-G) than during polymer assembling along silver-free P(MEO_2_MA) nanogels formation (cur-G).

**Figure 4:**
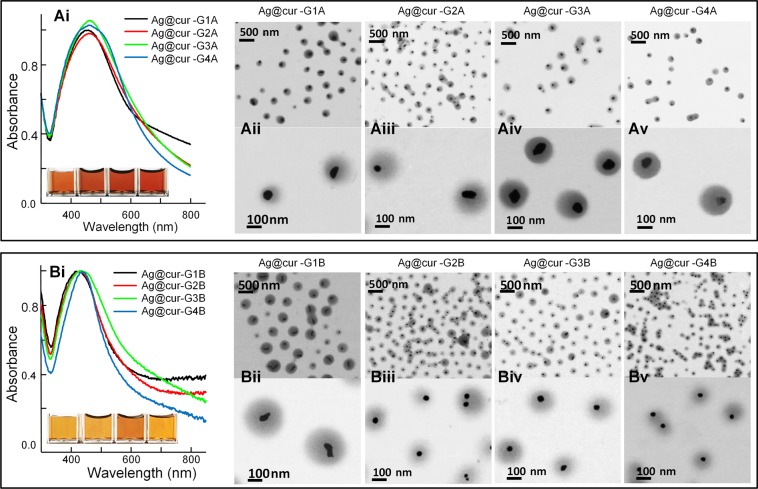
UV-Vis spectra and representative Bright Field S-TEM images of Ag@cur-P(MEO_2_MA) core-doped shell hybrid NPs with AgNP cores synthesized at: (**A**) 90 °C, Ag@cur-G#A series (Table 1) and (**B**) 100 °C, Ag@cur-G#B series (Table 1). Insets show color variation of the samples.

S-TEM micrographs in Fig. 4 evidence a core-shell morphology of the Ag@cur-G hybrid NPs with silver core formed at 90 °C (series Ag@cur-G#A, Fig. 4A) and at 100 °C (series Ag@cur-G#B, Fig. 4B). A mere glance to S-TEM pictures revealed almost concentric nanostructuration of polymer shell coverage around metal core for both series of nanohybrids. Both series were synthesized with similar curcumin/polymer ratios (3.80, 3.15, 2.10 and 1.05 wt%, Table 1). The resultant mean values of Ag-core diameter (60 nm at 90 °C and 40 nm at 100 °C) for both series are collected in Table 1. These differences can be attributed to the faster nucleation rate with increasing temperature that leads to smaller NPs. EDX spectra in Fig. S3 confirm the presence of Ag in two representative hybrid samples with 60 nm (Ag@cur-G4A) and 40 nm (Ag@cur-G4B) core diameter, the determined elemental composition is in agreement with ther core size of both nanohybrids.

Moreover, it seems that samples synthesized with the two highest curcumin ratios (3.15 wt%, Fig. 4Aiv-Biv and 3.8 wt%, Fig. 4Av-Bv) exhibit a more homogeneous spherical shape and a quasi monodistribution, especially, when synthesized at 100 °C (Fig. 4Biv-v). This is in contrast to what happens with the lowest curcumin concentration (Fig. 4Aii and Bii) where two silver NP populations appeared during the synthesis.

These morphological changes were found to affect the SPR of the resulting core-doped shell Au@cur-G hybrid NPs. SPR bands of Ag@cur-G#A samples synthesized at 90 °C (UV-Vis, Fig. 4Ai) are broader and red-shifted compared to those of Ag@cur-G#B hybrid NPs obtained at 100 °C (UV-Vis, Fig. 4Bi). In fact, these wavelengths variations resulted from changes of refractive index of the medium surrounding the metal surface, due to polymer/curcumin reorganization around the two different Ag-core sizes. Correlative changes in color were also observed when different sizes of metal core were used by keeping all the other reaction parameters constant (Table 1 and UV-Vis insets of Fig. 4). Larger silver NPs exhibited a visual reddish color and a wavelength red-shift. In fact, the *λ*_max_ for Ag@citrate of 40 and 60 nm are 412 and 431 nm respectively^36^, so that red shifts of about 20–30 nm collected in Table 1 are in accordance to the polymer coating with shell thickness values shown in Table 1.

Aware of the importance of chemical variables, as curcumin and citrate reducing agents, on the silver-nuclei and their impact on the polymer-growth, an additional experiment with a higher amount of sodium citrate was programmed. At this time, 800 μL of a citrate solution 0.12 M was added to the reaction mixture instead of 0.029 M, in the presence of 3.8 wt% of curcumin, at 100 °C. As expected, it resulted in two highly differentiated Ag-core populations (30 nm and 5 nm) shown in Fig. S4. Interestingly, AgNPs (∼5 nm) were mostly formed at the limit around the polymeric vesicle boundary but also closely packed inside polymer networking-shell of 250 nm of size (Fig. S4). This behavior is attributed to the high nucleation of the nanoparticles due to the high concentration of citrate that causes a greater ion exchange and increases the total ionic strength in the solution, promoting the formation of particle agglomerates in some cases. Then, in the regime of low curcumin concentration, phase separation can take place, being the smallest spherical cores produced by sodium citrate and the almost rod-like AgNPs promoted by curcumin. So, we hypothesize that an appropriate balance among both reducing agents (sodium citrate and curcumin) is needed to obtain more uniform AgNP population.

### Thermoresponsive Properties

**Figure 5:**
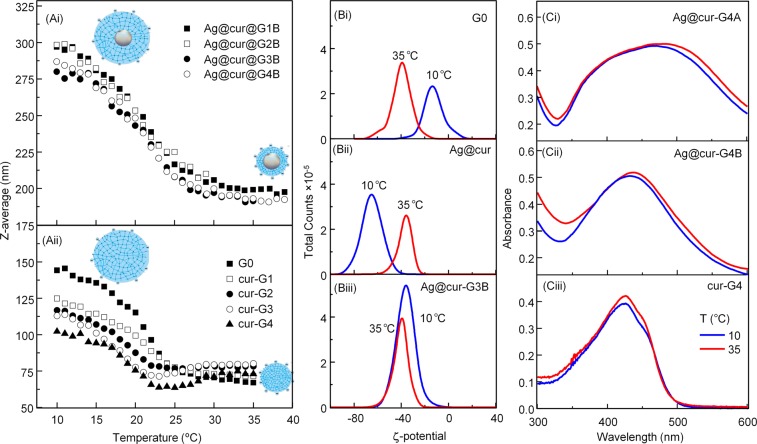
(**A**) Temperature-dependent hydrodynamic diameter evolution in water of (Ai) Ag@cur-P(MEO_2_MA) B-series core-doped shell hybrid NPs (Table 1) and (Aii) cur-P(MEO_2_MA) nanogels (Table 2) with different curcumin content. (**B**) ζ-potential determined by DLS at 10 °C and 35 °C for colloidal samples of (Bi) P(MEO_2_MA) nanogels (G0), (Bii) AgNPs functionalized with 3.15 wt% curcumin content (Ag@cur) before and (Biii) after (MEO_2_MA) polymerization, Ag@cur-P(MEO_2_MA) hybrid NPs (Ag@cur-G3B). (C) UV-Vis spectra (below and above VPTT) of Ag@cur-P(MEO_2_MA) hybrid NPs with Ag core synthesized at 90 °C (Ci) and 100 °C (Cii), and cur-P(MEO_2_MA) nanogel (Ciii). All samples shown (**C**) were synthesized with 3.8 wt% curcumin/polymer ratio (Table 1 and Table 2).

Micro/nanogels of crosslinked P(MEO_2_MA) in aqueous solution exhibit a reversible volume decrease when raising temperature above the lower critical solution temperature (LCST) of linear P(MEO_2_MA). This temperature is known as temperature-induced volume phase transition (VPTT) which can be followed by measuring the particle hydrodynamic size by DLS. In Fig. 5 representative Z-average *vs* temperature curves are shown for the series of Ag@cur-G#B NPs synthesized at 100 °C (Fig. 5Ai and Table 1) and of the cur-G# nanogels (Fig. 5Aii and Table 2). As can be observed the VPTT values, calculated from the inflection point of the curve (Fig. 5Ai), are around 20 °C for the two hybrid NPs series (Ag@cur-G, Table 1), showing no dependence on the curcumin content. These results are in good agreement with the ATR-FTIR spectra (Fig. 3) which indicated rather similar curcumin content for Ag@cur-G hybrids NPs. On the contrary, cur-G nanogels (Fig. 5Aii) exhibit slightly decreasing VPTT values with increasing the curcumin content (Table 2). This implication is clearly evidenced at low temperature, where the decrease of the nanogel size is affected by the hydrophobicity feature of curcumin (Fig. 5Aii).

Interestingly, hybrid nanoparticles exhibit a higher NPs size than polymeric nanogels. A plausible explanation of this behavior can lie on the impact of metal core which acts as seed during the precipitation-polymerization; since hybrid NPs may need to grow more in order to decrease the polymer-metal interfacial energy and to reach then the stabilization.

Swelling ratio (*Q*) is an important parameter affecting nanogel properties that can be determined by DLS. It can be directly calculated from the ratio between the nanogel volume (*V*) at swollen state and at the collapsed state^14^:

3 $$Q=\frac{V(swollen)}{V(collapsed)}$$

While for polymeric nanogels the volume is directly calculated from the particle radius (*R*) as *V* = 4/3π *R*^3^, for spherical hybrid NPs, shell volumes at both temperatures are calculated as *V*(shell) = 4/3π [*R*_p_^3^ – *R*_c_^3^]. *R*_P_ is the hydrodynamic radius of the hybrid particle and *R*_c_ the hydrodynamic radius of the Ag core. Table 1 shows the swelling ratios for the silver-polymer (Ag@cur-G) hybrid NPs and Table 2 for the polymeric (cur-G) nanogels investigated.

The observed temperature-responsive swelling ratios (*Q*) were close to 3 for the most of the hybrid NPs and slightly higher for polymeric nanogels. Indeed, for polymeric nanogels (Table 2) a decrease of *Q* results from the increase of curcumin in the polymeric nanogels, which could be attributed to a lower swelling ability since the hydrophobic balance increases. On the other hand, the fact that for Ag@cur-G hybrid NPs, *Q* values show no dependence on the feed composition, could indicate that the final composition of the polymer P(MEO_2_MA) shell is rather similar, corroborating again the ATR-FTIR results (Fig. 3D).

Some cyclic heating-cooling experiments were done (data not shown), after observing the reversible collapse-swelling behavior of the samples (Fig. 5). At low temperature, the steric protection by the hydrophilic P(MEO_2_MA) shell provides good stability in the aqueous medium; whereas at higher temperature, the driving force of colloidal stability is the electrostatic repulsion among the particles revealed by *ζ*-potential measurements. Tables 1 and 2 show *ζ*-potential values corresponding to Ag@cur, Ag@cur-G hybrid NPs and cur-G nanogels at 35 °C. All types of NPs synthesized with curcumin exhibit a fairly high negative *ζ*-potential that would confer stability against aggregation, especially at temperature above the VPTT, where the increase of hydrophobic balance in the polymer shell suddenly produces water expelling from the nanogel.

The negative *ζ*-potential of the synthesized NPs can be attributed to several origins. It is well well-known that during polymerization process, incorporation of initiator groups (anionic persulfate) into polymer chains introduces negative charges into micro/nanogel particles synthesized by FRPP. Additionally, in the specific synthesis of the nanohybrids with silver core explored in this work, the contribution of the remanent sodium citrate cannot be neglected. Even more, the negative *ζ*-potential may arise from curcumin, as it has been reported by some authors^19,37^.

Certainly, in previous works, we found an increase of the *ζ*-potential at temperature above polymer collapse for both, thermoresponsive polymeric nanogels and hybrid NPs with metallic Au-core^14^. This effect was attributed to the decrease of the particle size, which increases the charge density at the NP surface. Nevertheless, in the present investigation, we only observed this temperature-dependent effect on nanoparticles without curcumin (G0 in Fig. 5Bi). Indeed, hybrid NPs exhibited a tunable *ζ-*potential temperature-dependent behavior before (Ag@cur) and after polymerization (Ag@cur-G). So, initial *ζ-*potential diminution values with increasing temperature (Fig. 5Bii) rapidly transited to high and almost invariant *ζ*-potential values with temperature (Fig. 5Biii) as polymerization progressed. These observations (Table 1 and Fig. 5B) so far can be explained through the antagonistic tendencies of the polymer shell and curcumin.

The ability of the polymer shell to swell or collapse as function of temperature can tune the optical and luminescent properties of the synthesized nanoparticles. Figure 5C displays representative UV-Vis spectra (below and above VPTT) for two nanohybrid samples with different core size (Ag60nm@cur-G4A, Fig. 5Ci and Ag40nm@cur-G4B, Fig. 5Cii) and nanogels (cur-G4, Fig. 5Ciii) synthesized with 3.8 wt% of curcumin content (Table 1). In UV-Vis absorbance spectra, SPR band of the nanohybrid with a higher silver diameter (Fig. 5Ci) red-shifted while broadening considerably with respect to SPR band of the nanohybrid with a smaller silver diameter (Fig. 5Cii). Obviously, the SPR band position and width was affected by the core-size and the temperature-induced variation of the electromagnetic field in its neighboring environment. The cur-G4 sample containing silver-free nanogel (Fig. 5Ciii) only showed the curcumin effect on nanogel swelling behavior which determines the refractive index of the polymer-gel with the surrounding aqueous medium. In addition, a reversible red-blue shift was observed, when the temperature increase-decrease the VPTT of the polymer shell (Fig. 5). When the external temperature rises above the VPTT, the nanogel shell expels water dictated by the strengthening of hydrophobic interactions giving rise to (i) a decrease of the polymer shell thickness and (ii) an increase of the refractive index. At that point, the effect of refractive index augmentation exceeds to that of the shell thickness diminution; therefore a red-shift should be observed above the VPTT for plasmonic-core@thermoresponsive-shell nanohybrids. Fig. 5Ci and 4Cii illustrate this behavior for our Ag-based thermoresponsive-polymer (core@shell) hybrid NPs which correlates well with similar results reported for Au-core based thermoresponsive core@shell nanogels^38^.

Furthermore, the change of the SPR maximum with temperature is more pronounced for larger silver core (Fig. 5Ci) as previously observed for core-shell nanohybrids with gold core^15^. Indeed for the nanohybrids with silver core of 60 nm (series Ag@cur-G#A), the ΔλLSPR_max_ is about 6–10 nm whereas for the 40 nm silver core ones (series Ag@cur-G#B) the ΔλLSPR_max_ is about 2–4 nm (Table 1). The cur-G4 nanogel sample, without metallic core intervention (Fig. 4Ciii), exhibits no so remarkable λ_max_ absorption change with temperature, at similar curcumin/polymer ratio.

### Luminescent Properties

Curcumin is a tautomeric compound that can occur in diketo and keto-enol forms which ratio strongly varies with the solvent. According to Manolova *et al*.^39^, in solvents as ethanol, only the enol–keto tautomer is present; whereas the addition of water leads to appearance of a new spectral band (about 350 nm), which was attributed to the diketo tautomeric form. The keto-enol form, due to a strong intramolecular hydrogen bond, undergoes transformation into a totally delocalized π-system. Due to the hydrophobic character of curcumin, it presents luminescence properties in organic solvents but it is almost non-fluorescent in aqueous solution. However, by complexation through hydrophobic interactions with proteins^40^ or with amphiphilic polymers as pluronic^41^, or encapsulating curcumin in hydrophobic polymer nanoparticles^30^, fluorescence enhancement in aqueous medium has been reported. Previous to the luminescence investigation of the Ag@cur-G hybrid NPs, we investigated the absorption and emission of curcumin encapsulated in the thermoresponsive nanogels, cur-G (Table 3 and Fig. S5). Hydrophobic curcumin was engulfed by thermoresponsive NPs during the polymerization process, based on the precipitation of the growing polymer at 70 °C due to its unfavorable interaction with the water solvent above LCST (Fig. 1). Absorbance spectra for curcumin embedded in P(MEO_2_MA) nanogels (cur-G) at the polymer concentration of 1 mg mL^−1^ show an increase of intensity proportional to the curcumin concentration (Fig. S5). In agreement with previous reports of curcumin entrapped in hydrophobic^30^ or amphiphilic block copolymers^41^, a main absorption band at 425–430 nm in addition to a shoulder about 450 nm^30^ appear in Fig. S5. Some authors attributed the signal at 430 nm to the keto-enol tautomer^39,42^, whereas the band at 350 nm, associated to the keto form and that typically appears in water, is absent in the present case. Fig. S5 inset shows the linear dependence of the curcumin absorption maxima at 427 nm *vs* molar concentration of curcumin according to the Beer-Lambert law for cur-G nanogels.

**Table 3:** Luminescent properties of curcumin encapsulated in the thermoresponsive nanogels

Entry	Curcumin^a^ wt%	*λ*_em_^b^	*ϕ*_F_		
			10 °C	22 °C	50 °C
cur-G1	1.05	509	0.055	0.077	0.147
cur-G2	2.10	512	0.027	0.040	0.065
cur-G3	3.15	515	0.029	0.042	0.084
cur-G4	3.80	514	0.022	0.036	0.045
Ag@cur-G1A	1.05	508	0.013	0.013	0.014
Ag@cur-G2A	2.10	504	0.016	0.017	0.017
Ag@cur-G3A	3.15	500	0.017	0.021	0.018
Ag@cur-G4A	3.80	502	0.028	0.032	0.030
Ag@cur-G1B	1.05	508	0.014	0.014	0.015
Ag@cur-G2B	2.10	516	0.018	0.019	0.017
Ag@cur-G3B	3.15	515	0.024	0.027	0.023
Ag@cur-G4B	3.80	514	0.013	0.016	0.014

^(a)^Curcumin/polymer ratio, ^(b)^ *λ*_exc_ was 430 nm

**Figure 6:**
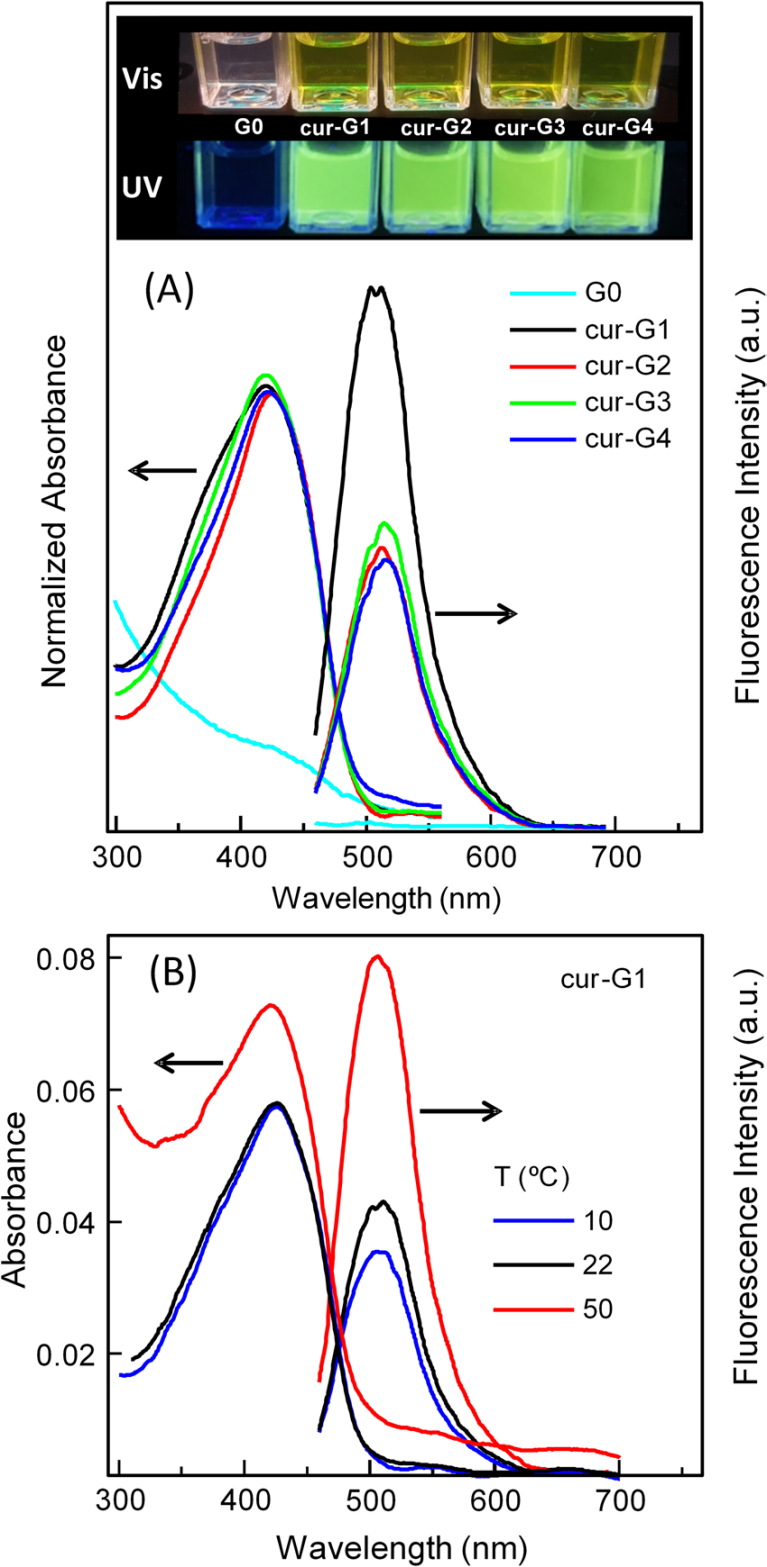
(**A**) Absorption and emission spectra corresponding to a series of cur-P(MEO_2_MA) nanogels (cur-G#, Table 2) in water at 22 °C. The inset shows samples reproduced in (**A**) under visible and UV light. (**B**) Temperature dependence of the emission of cur-P(MEO_2_MA) nanogel with 1.05 wt% curcumin content (cur-G1 sample in Table 3). Samples were excited at 430 nm.

In Fig. 6A emission spectra in water for the synthesized cur-G nanogels excited at 430 nm evidence remarkable luminescence due to curcumin. In Table 3, the emission maxima (*λ*_em_) and QY, calculated with fluorescein standard, are collected. The emission maxima are about 509–515 nm in good agreement with the values reported by Banerjee *et al*.^30^ for curcumin encapsulated in polymeric nanoparticles. The position of the emission maxima of curcumin is strongly dependent on the solvent; so by increasing the polarity from chloroform to water, the emission maximum is red-shifted and broadened from 497 nm to 550 nm^42^. Both the QY values and the emission maxima position for the present cur-G nanogels seem to indicate that curcumin is well protected by the hydrophobic environment provided by the polymer.

With respect to the QY values, two facts have been observed: first, the QY value increases with the decrease of curcumin concentration in the nanogels (Fig. 6A and Table 3); and, second, an amazing fluorescence enhancement is noticed by the temperature increase (Fig. 6B).

The effect of curcumin concentration could be related to the protection that hydrophobic domains of the polymer offer to curcumin, which is enhanced at low curcumin/polymer ratio. The increase of luminescence with increasing temperature should be associated to the water expelled out of the nanogels when temperature rises above VPTT. Indeed, at lower curcumin ratio (Fig. 6B), the remarkable increase in absorbance at 50 °C can be attributed to the higher polymer contribution. This reversible change in luminescence with temperature has been previously reported for fluorescent dyes as BODIPY in thermoresponsive P(MEO_2_MA) linear polymers and hydrogels^43^.

**Figure 7:**
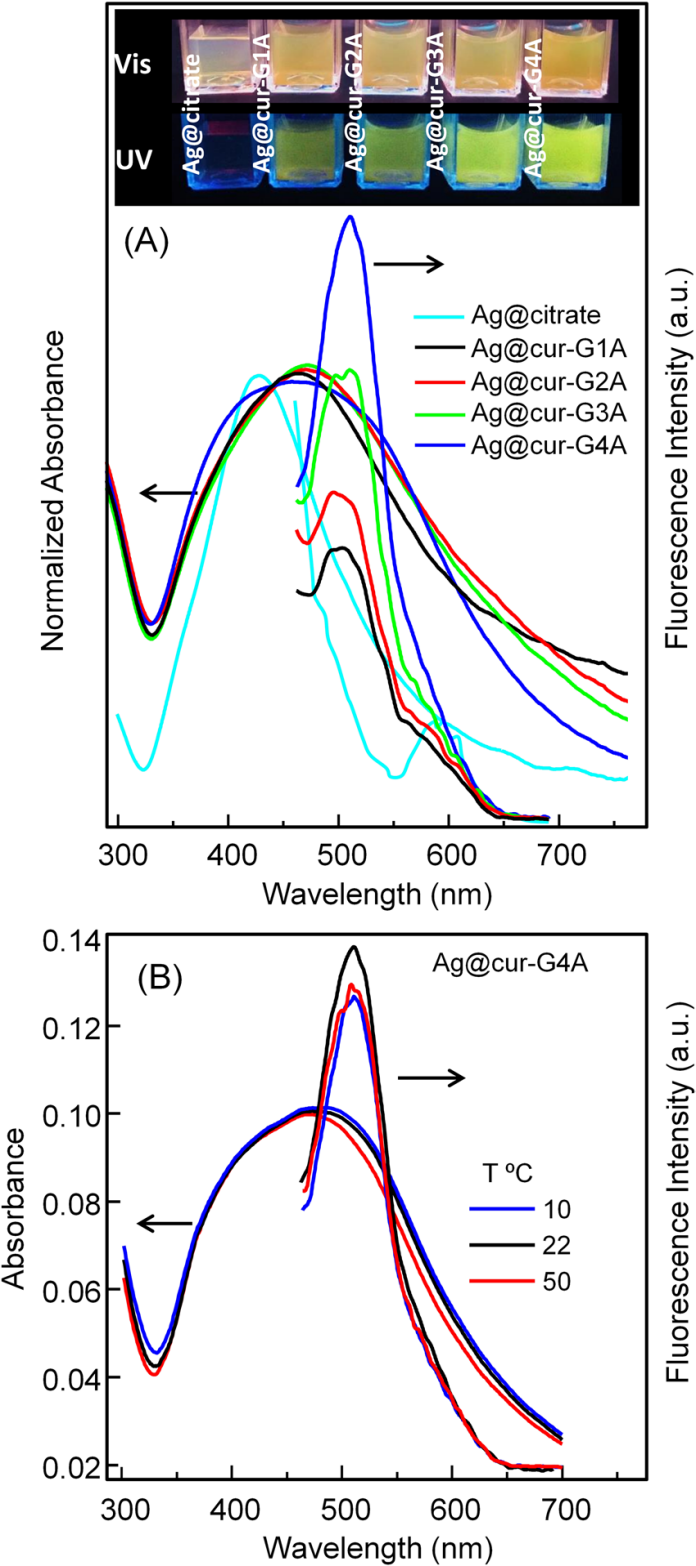
(**A**) Absorption and emission spectra corresponding to a series of Ag@cur-P(MEO_2_MA) core-doped shell hybrid NPs (Ag@cur-G#A, Table 1) in water at 22 °C. The inset shows samples reproduced in (**A**) under visible and UV light. (**B**) Temperature dependence of the emission of the Ag@cur-P(MEO_2_MA) nanohybrid with 3.8 wt% curcumin content (Ag@cur-G4A sample in Table 3). Samples were excited at 430 nm.

In Fig. 7 and Fig. S6 a different behavior can be found for hybrid NPs. In first place, analyzing the QY values collected in Table 3, it can be seen that nanohybrids´ fluorescence values are lower than those measured for cur-G nanogels. On the other hand, for both series of Ag@cur-G hybrid NPs, an increase of luminescence with increasing of curcumin concentration is detected, in opposite to what happened in cur-G nanogels (Table 3 and Fig. 6A). However we know that it can be an ¨apparent increase¨ since the absorption band consideration for estimating the QY values was not properly drawn up. Note that this absorption band reflects the additive contribution from the overlapped silver and curcumin SPR bands. Then, on the basis of these considerations, at lower curcumin concentration, the real curcumin contribution to the final absorbance will be lower. The same miscalculation could arise when comparing QYs of hybrid NPs with those of polymeric nanogels, because for hybrids an important part of the absorbance arises from the SPR of AgNPs.

In last place, the temperature effect also departs from that effect observed for cur-G nanogels, where an increase of fluorescence occurred after polymer collapse on account of hydrophobicity augmentation. In the case of hybrid NPs, a slight decrease of fluorescence is detected by moving away from room temperature. This experimental evidence suggests a change on the curcumin-polymer interaction due to the silver presence, or in this specific case, that the interaction of curcumin with silver played a major role, since the specific silver-nucleation strategy causes that certain amount of curcumin be confined at the metal surface.

The fluorescence diminution that accompanies the temperature decrease may find its origin in the hydrophilicity-gain experienced by the thermoresponsive polymer shell and the subsequent quenching of curcumin emission induced by water. Whereas, when increasing temperature, the polymer increases its hydrophobic balance, which should contribute to protect curcumin against water; nevertheless this positive effect could be overcome by the fact that after polymer collapse, curcumin can get too close to the metal surface and this effect should lead to a further emission quenching. Similar observation was reported on the strong curcumin emission-quenching for other silver NP-based hybrid systems by other authors^19,20^.

## Conclusion

In this work, we focused on the knowledge and application of *one-pot* protocol to obtain core-shell hybrid nanoparticles with the ability to encapsulate hydrophobic, bioactive, antioxidant, and low bioavailability molecules. The aim is to draw on the reductive capacity of some bioactive molecules as curcumin for the *in situ* formation of metallic NPs, meanwhile their hydrophobic character leads the polymer nanostructuration in aqueous medium in the presence of the inorganic metal-core. Therefore, new core-doped shell nanohybrids based on silver plasmonic core, thermoresponsive polymer shell and embedded luminescence and bioactive curcumin are easily obtained with potential bioapplications such as antimicrobial systems. Furthermore, polymeric nanogels encapsulating curcumin were also achieved, which luminescent properties are strongly enhanced by increasing temperature. This last property could be tuned even with the minimum ratio of bioactive compound.

## Supplementary information

Supplementary Info
