# Supplementary material for: Curcumin to Promote the Synthesis of Silver NPs and their Self-Assembly with a Thermoresponsive Polymer in Core-Shell Nanohybrids
Source: Sci Rep. 2019 Dec 3;9:18187. doi: 10.1038/s41598-019-54752-4 (PMC6890765; doi:10.1038/s41598-019-54752-4)
Supplement: Supplementary file 1 — Supplementary Info [file 41598_2019_54752_MOESM1_ESM.docx]

Supplementary Information

Curcumin to Promote the Synthesis of Silver NPs and their Self-Assembly with a Thermoresponsive Polymer in Core-Shell Nanohybrids

Albanelly Soto-Quintero, Nekane Guarrotxena,* Olga García and Isabel Quijada-Garrido*

Group of Nanohybrids and Interactive Polymers (NyPI), Instituto de Ciencia y Tecnología de Polímeros. Consejo Superior de Investigaciones Científicas (ICTP-CSIC), c/ Juan de la Cierva, 3. E-28006 Madrid, Spain.

N.G.: E-mail: [nekane@ictp.csic.es](mailto:nekane@ictp.csic.es); Tel: (+34) 915622900

I.Q-G.: E-mail: [iquijada@ictp.csic.es](mailto:iquijada@ictp.csic.es); Tel: (+34) 915622900

**List of Contents**

ATR-FTIR spectra of trisodium citrate, Ag@citrate, curcumin, Ag@cur NPs, Ag@cur-G3B NPs and P(MEO_2_MA) NPs (Figure S1)………………………………………………………………………………….….....2

**Bright Field S-TEM image and DLS measurement particle-size of hybrid nanogels with citrate-stabilized silver core synthesized without curcumin intervention**

**(Figure S2) ……………………………………………………………………………………………….……………2**

**EDX spectra for Ag@cur-G4A nanohybrid with 60 nm Ag core and for Ag@cur-G4B nanohybrid with 40 nm Ag core (Figure S3)………………………………………………………………………………………………….3**

Bright field S-TEM images of hybrid nanogels synthesized with curcumin and excess of trisodium citrate (Figure S4) ……………………………………….……………….…………………………...…………...….………4

Absorption spectra of a series of cur-P(MEO_2_MA) NPs with different curcumin concentration (Figure S5)……...……………………………………………………………….………….………………………...…..........4

Absorption and emission spectra of Ag@cur-GB# core-doped shell nanohybrids (Figure S6) …………………………………………………………………………………………………………….….…………5


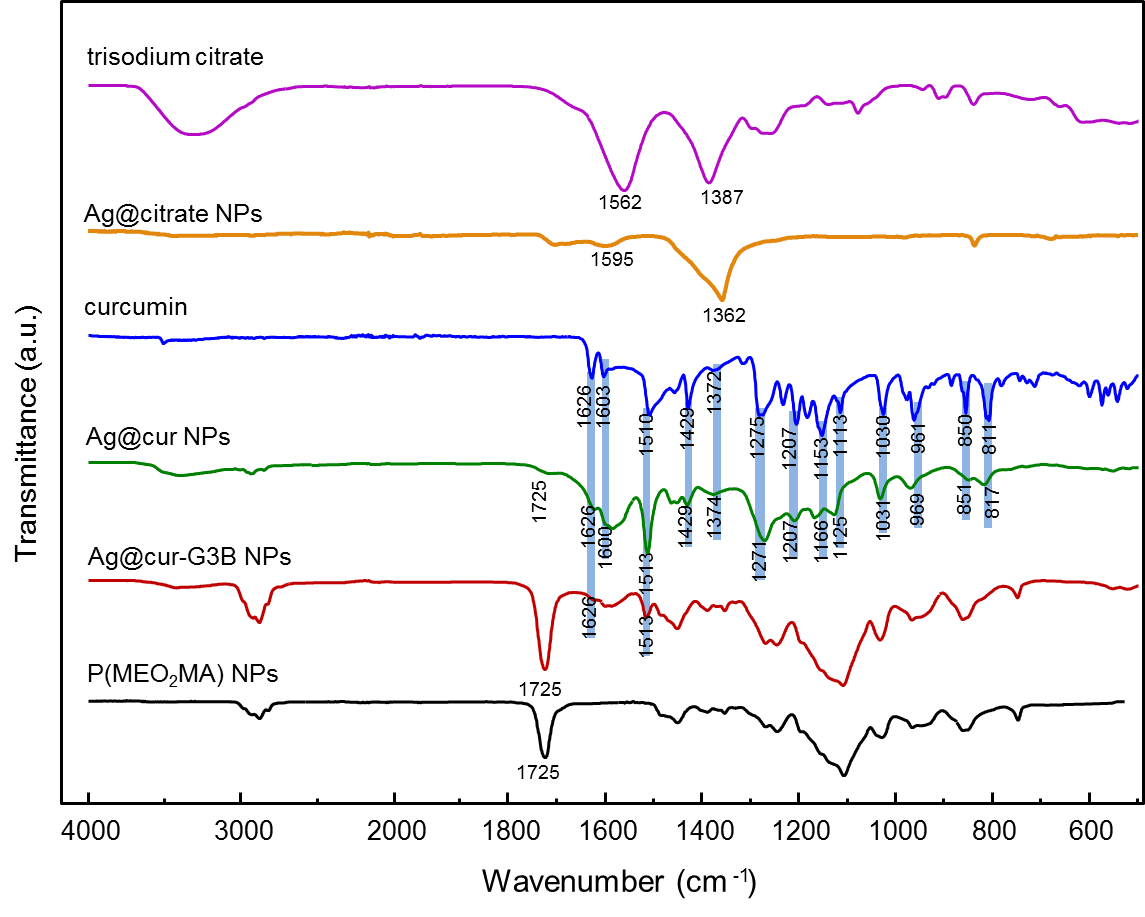


**Figure S1.** ATR-FTIR spectra corresponding to trisodium citrate (violet line); silver capped with citrate, Ag@citrate NPs (orange line); curcumin (blue line); silver NPs decorated with curcumin, Ag@cur NPs (green line); silver NPs decorated with curcumin and encapsulated with P(MEO_2_MA) nanogel, Ag@cur-P(MEO_2_MA) NPs (red line); and raw MEO_2_MA-based polymer NPs, P(MEO_2_MA) NPs (black line).


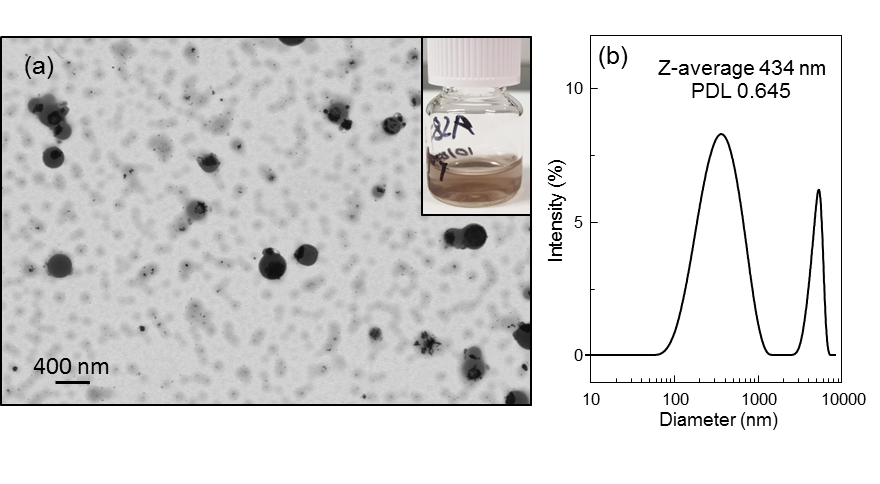


**Figure S2.** (a) Representative Bright Field S-TEM image of a hybrid nanogel with citrate-capped silver core synthesized without curcumin intervention reveals the presence of polymer NPs and large AgNPs aggregates. In the inset picture, the grey color of the solution corroborates the formation of aggregate. (b) DLS measurement of size distribution by intensity corresponding to sample shown in (a). The multimodal distribution and the high polydispersity (PDL = 0.645) of the sample corroborates the presence of aggregates.

**Figure S3.** Representative EDX spectra of hybrid nanogels with silver core synthesized in the presence of curcumin: (a) EDX spectrum for Ag@cur-G4A nanohybrid with 60 nm Ag core and (B) EDX spectrum for Ag@cur-G4B nanohybrid with 40 nm Ag core.


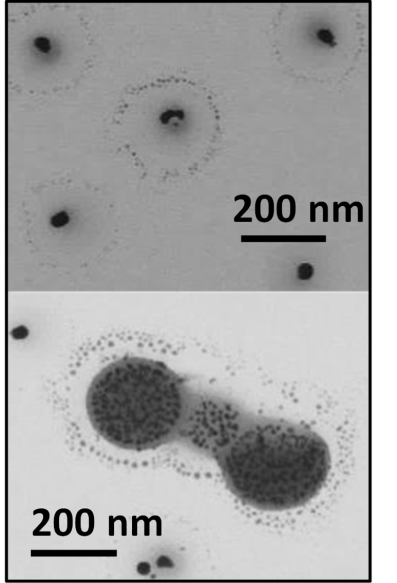


**Figure S4.** Representative Bright Field S-TEM images of hybrid nanogels with silver core synthesized in the presence of curcumin and excess of trisodium citrate.


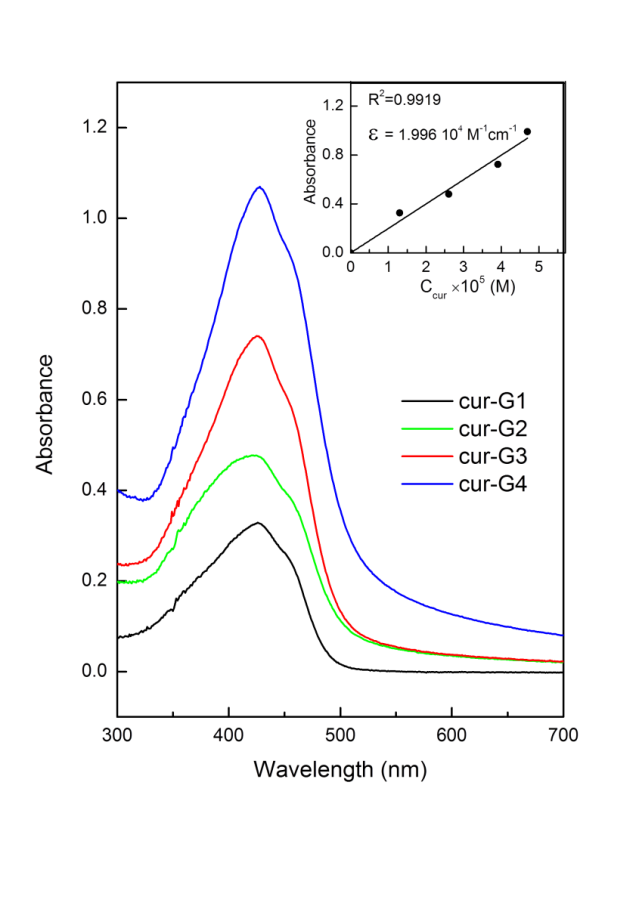


**Figure S5.** Absorption spectra of a series of cur-P(MEO_2_MA) NPs with different curcumin concentration from 3.8, 3.15, 2.10 and 1.05 wt% curcumin/polymer, at 1 mg mL^-1^ polymer concentration, in water (cur-G#, Table 2). The inset shows linear relation of the absorption intensity at 427 nm *vs* curcumin molar concentration in this concentration range. The molar attenuation coefficient (*ε*) of curcumin encapsulated in P(MEO_2_MA) NPs according to the Beer-Lambert law is also given.


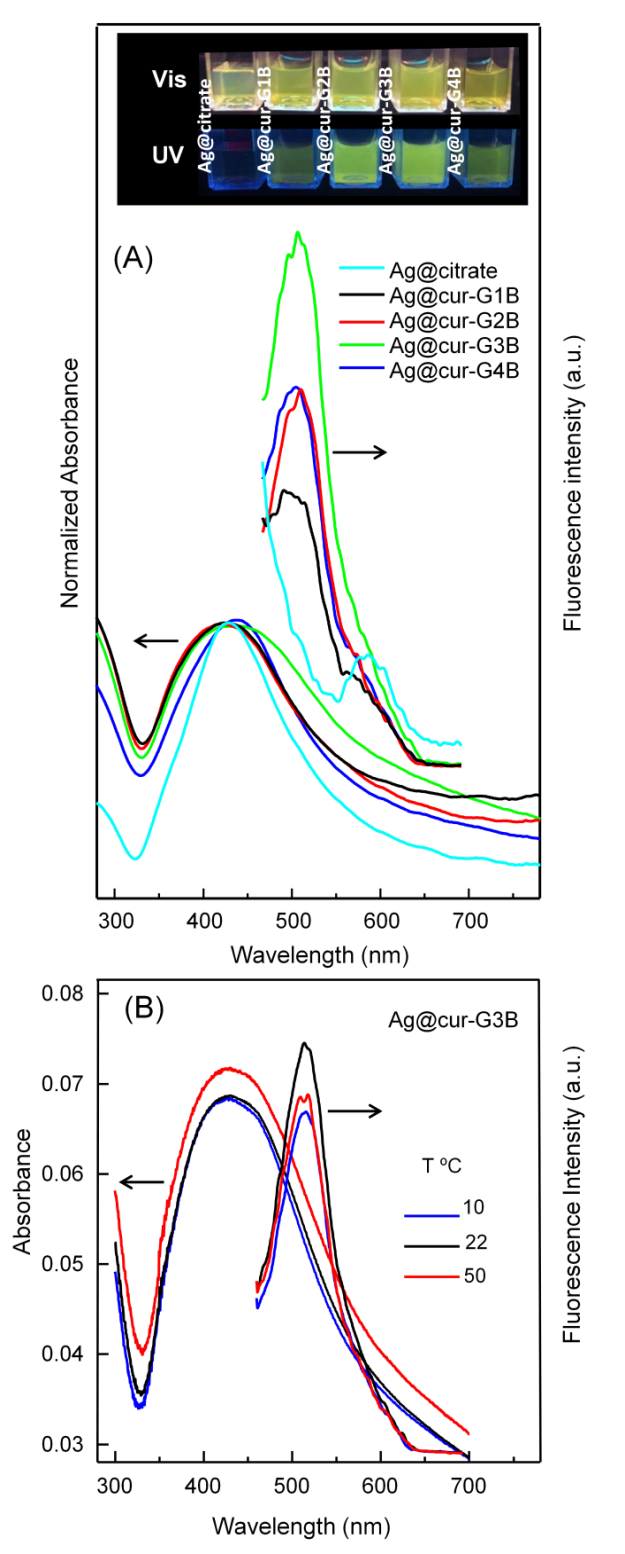


**Figure S6.** (A) Absorption and emission spectra corresponding to a series of Ag@cur-P(MEO_2_MA) core-doped shell nanohybrids (Ag@cur-G#B, Table 1) in water solution at 22 ºC. The inset shows samples reproduced in (A) under visible and UV light. (B) Temperature dependence of the emission of Ag@cur-P(MEO_2_MA) nanohybrid with 3.15 wt% curcumin content (Ag@cur-G3B sample in Table 3). Samples were excited at 430 nm.
